# Investigating the Triaxial Mechanical Behaviour of Silicone Rubber Material

**DOI:** 10.3390/polym18060755

**Published:** 2026-03-20

**Authors:** Jie Yang, Nan Chen, Jun Gao, Yang Wang, Shuchang Long, Xiaohu Yao, Zhibin Wu, Junfeng Zhao

**Affiliations:** 1 Shanghai Aircraft Design and Research Institute, Shanghai 201210, China; yangjie9@comac.cc (J.Y.);; 2 School of Civil Engineering and Transportation, South China University of Technology, Guangzhou 510640, China; 3 School of Aeronautics, Northwestern Polytechnical University, Xi’an 710072, China

**Keywords:** silicone rubber, biaxiality ratio, modelling, experiment, simulation

## Abstract

Silicone rubber is extensively used in engineering applications due to its toughness and impact resistance; however, traditional characterisation methods fail to capture its nonlinear deformation characterisation and triaxial mechanical behaviour. To address this, we derived a constitutive model within the framework of continuum mechanics that assumes a condition of near incompressibility and conducted uniaxial, planar, and equibiaxial tension tests to fit the model parameters. Through systematic analysis of triaxial mechanical responses under these three loading modes, we determined the material’s nonlinear large-deformation behaviour and sensitivity to the biaxiality ratio. Comparative analyses with classical hyperelastic models show that the proposed model achieves a good balance between the number of parameters and fitting accuracy. After the parameter-fitting process, we performed finite element simulations of the three loading modes. The simulation results show good agreement with experimental data in terms of deformation patterns and stress–strain curves. This study provides a novel theoretical tool for evaluating the mechanical properties and structural designs of soft materials.

## 1. Introduction

Elastomeric polymers exhibit extensive application prospects in the fields of transportation, security, medical health, sports, and many others due to their exceptional mechanical properties, including high elasticity (up to 100% or even 1000%), toughness, and impact resistance [1,2]. Of these, silicone rubber has also become an ideal material for numerous practical applications, such as soft robotics [3] and flexible energy harvesters [4,5], due to its high extensibility and durability, low dissipation behaviour, and biocompatibility. These practical applications require the material to withstand deformations under complex mechanical states, highlighting the need to predict silicone rubber’s complex mechanical behaviour. Studies have demonstrated that silicone materials exhibit significant sensitivity to the biaxiality ratio μ [6]. This variable is defined as $\mu =\frac{\ln\left(\lambda_{\min}\right)}{\ln\left(\lambda_{\max}\right)}$, where $\lambda_{\max}$ and $\lambda_{\min}$ represent the maximum and minimum principal stretches, respectively, and serves as a parameter characterising loading conditions and reflecting the degree of inhomogeneity in the deformation states experienced by materials. Biaxial mechanical behaviour is an important research direction in materials science and engineering, as it focuses on investigating material responses under different deformation modes.

In recent years, many studies have been conducted on the mechanical behaviour of silicone rubber materials. Liao et al. [7] performed systematic experiments to decouple the material’s mechanical response characteristics, revealing that it exhibits weak viscoelastic characteristics but significant stress softening and recovery behaviours. Kumar et al. [8] investigated the static and dynamic mechanical properties of polydimethylsiloxane (PDMS) under uniaxial tensile conditions, examining its viscoelastic behaviour under varying strain rates and force-controlled cyclic loading. However, the existing studies provide limited descriptions of mechanical behaviour under varying biaxiality-ratio conditions. Some researchers have also developed biaxial tensile testing systems for biomaterials, with PDMS materials used to validate the equipment’s accuracy and stability, e.g., Roth et al. [9] and Corti et al. [10]. Gao et al. [11] studied the mechanical behaviour of knee joint cartilage under biaxial cyclic loading conditions and characterised the associated ultrastructural changes. Their findings indicated that constraints in the Y-direction (orthogonal direction) could effectively reduce the maximum tensile strain in the X-direction. Liao et al. [6] performed cyclic loading–unloading tests on commercial silicone rubber under various deformation modes (uniaxial, planar, and equibiaxial tension) across multiple strain levels, revealing the material’s mechanical behaviour and stress recovery characteristics under repeated loading. Luo et al. [12] employed finite element analysis to evaluate the accuracy and comparability of three equibiaxial stretching methods: inflation stretching, equibiaxial plane stretching, and radial stretching.

Research on the mechanical properties of rubber-like materials continues to develop in tandem with theoretical frameworks of their physical constitutive relations. A central focus has developed: quantitatively characterising the intrinsic correlation between strain and stress in these materials while fully accounting for their critical mechanical characteristics, such as biaxiality-ratio sensitivity. Hyperelastic constitutive relations are a crucial theoretical framework for describing their nonlinear large-deformation mechanical behaviour. This theory posits the existence of a Helmholtz free energy function Ψ, which is a continuous scalar function dependent on the deformation gradient tensor F, capable of characterising the material’s energetic state during deformation processes. According to Holzapfel et al. [13], the strain energy function Ψ can be expressed through three independent invariants. Beyond uniaxial deformation, scholars have investigated constitutive models under various deformation modes. Ogden [14] demonstrated that the Neo-Hookean model [15] can only describe shear deformation in rubber within small-to-moderate strain ranges, whereas the Mooney–Rivlin model [16,17] incorporating the second invariant demonstrates broader applicability across diverse deformation modes. Xiao et al. [18] found that conventional isotropic damage models fail to capture the stress response of tough gels under multiaxial loading; they, therefore, developed a non-affine theory based on the microsphere model, which successfully predicted experimental results from pure shear and unequal biaxial tests in addition to damage cross-effects. Ostadrahimi et al. [19] proposed a physics-informed machine learning framework to simulate nonlinear, history-dependent viscoelastic mechanical behaviour under multiaxial cyclic loading conditions.

Based on these findings, we can summarise the current limitations in the field using the following categories: most experiments focus on uniaxial loading, with the use of systematic mechanical testing and characterisation techniques for complex triaxial stress states being relatively scarce; and existing models are mainly based on parameter fitting from uniaxial tests, neglecting the complex effects of biaxiality-ratio sensitivity inherent to elastometric polymers.

In-depth investigations, theoretical quantitative descriptions, and numerical implementations of the mechanical behaviour of silicone rubber, therefore, hold fundamental significance for effectively predicting the deformation, optimising the performance, and promoting the engineering applications of related materials.

This study focuses on the mechanical behaviour of silicone rubber under different deformation modes, aiming to thoroughly investigate the biaxiality-ratio-sensitive characteristics of related materials by developing a constitutive model. To achieve this objective, we designed and conducted uniaxial, planar, and biaxial tension tests; parameter fitting; and comparative analyses to evaluate the model’s accuracy regarding the nonlinear large deformation and biaxiality-ratio sensitivity of the material. Our results hold significance for enhancing precise simulation analyses of elastometric polymers under various deformation modes.

## 2. Constitutive Modelling

In this section, based on continuum mechanics, we develop a constitutive model to characterise the nonlinear large deformations and sensitivity-to-biaxiality ratio of the elastometric material.

### 2.1. Kinematics

**Figure 1:**
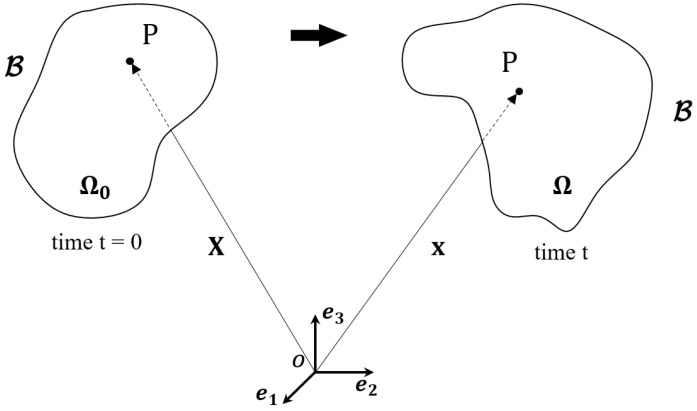
Configuration and motion of a continuum body B.

First, consider a continuum body B in three-dimensional Euclidean space, and introduce an initial coordinate system with the origin *O* and basis vectors $e_{i}\left(i=1,2,3\right)$, as shown in Figure 1. The continuum B undergoes motion from its initial configuration (reference configuration) $\Omega_{0}$ to the current coordinate system $\Omega_{t}$. When any material particle P moves from position $X\in \Omega_{0}$ to position $x\in \Omega_{t}$, the deformation gradient is given as

(1) $$F=\frac{\partial x}{\partial X}=F_{ij},\;i,j=1,2,3$$

The determinant of the deformation gradient,

(2) $$J\left(X,t\right)\overset{\mathrm{def}}{=}\det F=\frac{\partial v}{\partial V},$$

describes the volume change of the differential element, where *V* is the initial volume of the differential element, and *v* is the current volume. The right Cauchy–Green strain tensor is defined as

(3) $$C=F^{T}F$$

### 2.2. Clausius–Duhem Inequality

According to the Clausius–Duhem inequality [13], we have

(4) $$S:\frac{1}{2}\dot{C}-\dot{\Psi }-e\dot{\Theta }-\frac{1}{\Theta }Q\cdot \mathrm{Grad}\Theta \geq 0,$$

where S denotes the second Piola–Kirchhoff stress tensor; $\dot{C}$, $\dot{\Psi }$, and $\dot{\Theta }$ represent the rate forms of the right Cauchy–Green strain tensor, strain energy function, and temperature, respectively; *e* signifies internal energy; Q represents the heat flux vector; and GradΘ indicates the temperature gradient. The above equation establishes the thermodynamic relationship governing the mechanical response between stress and strain. Substituting the rate form of the strain energy function into Equation (4) yields

(5) $$\left(S-2\frac{\partial \Psi }{\partial C}\right):\frac{1}{2}\dot{C}-e\dot{\Theta }-\frac{1}{\Theta }Q\cdot \;\mathrm{Grad}\;\Theta \geq 0.$$

Considering the arbitrariness of $\dot{C}$, S can be written as

(6) $$S=2\frac{\partial \Psi }{\partial C}.$$

Similarly, the relationship between the first Piola–Kirchhoff stress tensor P and the strain energy function Ψ can be written as

(7) $$P=\frac{\partial \Psi }{\partial F}.$$

### 2.3. Nearly Incompressible Framework

Further studies have shown that elastomeric polymers exhibit a certain degree of compressibility. For example, in uniaxial tensile tests, vulcanised and pure rubber exhibit volume changes of 1% and 2%, respectively, at deformations of up to 700% [20,21]; in other deformations, the change is even lower [22]. Some softer materials, such as polyurethane and hydrogels, exhibit greater compressibility [23,24]. It is necessary to consider the nearly incompressible nature of elastomeric polymers; not only can this more accurately describe their mechanical behaviour (even slight volume changes can have a significant effect), but it can also alleviate numerical difficulties and instabilities caused by “incompressibility” in finite element simulations [25].

For nearly incompressible models, the strictly incompressible model is modified into an isochoric strain energy function with an added volumetric deformation part, known as the “penalty function” method [25]. In this framework, the deformation gradient is multiplicatively decomposed into volume-changing (dilational) and volume-preserving (distortional) parts, i.e.,

(8) $$F=J^{-1/3}\bar{F},$$

where $\bar{F}$ is the modified deformation gradient; its determinant can be derived as $\det\bar{F}=1$, which demonstrates the volume-preserving characteristic of this tensor. Under this decomposition approach, subsequent deformation analysis requires the use of the isochoric form of the right Cauchy–Green strain tensor, i.e.,

(9) $$\bar{C}=J^{-2/3}C.$$

In a nearly incompressible framework, the strain energy function of elastomeric polymers can be decomposed into isochoric and volumetric parts, i.e.,

(10) $$\Psi =\Psi_{\mathrm{iso}\;}\left({\bar{I}}_{1},{\bar{I}}_{2}\right)+\Psi_{\mathrm{vol}\;}\left(J\right),$$

where $\Psi_{\mathrm{iso}\;}\left({\bar{I}}_{1},{\bar{I}}_{2}\right)$ is the isochoric strain energy function, and

(11) $$\left\{\begin{array}{c} {\bar{I}}_{1}=\mathrm{tr}\left(\bar{C}\right)=\mathrm{tr}\left(J^{-2/3}C\right)=J^{-2/3}\mathrm{tr}\left(C\right)\\ {\bar{I}}_{2}=\frac{1}{2}\left[{\left(\mathrm{tr}\bar{C}\right)}^{2}-\mathrm{tr}\left({\bar{C}}^{2}\right)\right]\\ \bar{J}=1 \end{array}\right.$$

represents the isochoric principal invariants. Note that tr() in Equation (11) denotes the trace operation. In Equation (10), the volumetric strain energy function

(12) $$\Psi_{\mathrm{vol}}\left(J\right)=\frac{1}{D}{\left(J-1\right)}^{2}$$

is commonly referred to as the penalty function [25], which helps improve computational stability in numerical simulations. The parameter $\frac{1}{D}$ is related to the bulk modulus and is typically large for elastomeric polymers. Determining the volumetric parameter usually requires volumetric deformation experiments to be performed; however, research in this area is relatively limited. Ogden [22] suggested introducing a coupling strain energy term between the isochoric and volumetric parts to further improve the accuracy of the problem description. However, doing so removes the advantageous separability of the stress and elasticity tensors in such constitutive relations and increases the difficulty of determining the coupling term parameters. Therefore, for simplicity in problem handling, we adopt an additive decoupling approach for the strain energy function.

In Equation (6), the second Piola–Kirchhoff stress tensor can be similarly decomposed into isochoric and volumetric parts, i.e.,

(13) $$S=S_{\mathrm{iso}}+S_{\mathrm{vol}}.$$

Isochoric stress can be expressed as

(14) $$\begin{array}{c} S_{\mathrm{iso}\;}=2\frac{\partial \Psi_{\mathrm{iso}\;}}{\partial \bar{C}}:\frac{\partial \bar{C}}{\partial C}=J^{-2/3}\left(I-\frac{1}{3}C^{-1}⊗C\right):\bar{S}=J^{-2/3}P:\bar{S} \end{array}$$

and volumetric stress can be expressed as

(15) $$S_{\mathrm{vol}}=J\frac{\partial \Psi_{\mathrm{vol}}\left(J\right)}{\partial J}C^{-1}=JpC^{-1}.$$

To facilitate understanding of Equation (14), we explain the differential process of $\frac{\partial \bar{C}}{\partial C}$ below. Considering

(16) $$\frac{\partial J}{\partial C}=\frac{J}{2}C^{-1},$$

we have

(17) $$\begin{array}{c} \frac{\partial \bar{C}}{\partial C}=\frac{\partial \left(J^{-2/3}C\right)}{\partial C}=J^{-2/3}\left(I+J^{2/3}C⊗\frac{\partial J^{-2/3}}{\partial C}\right)=J^{-2/3}\left(I-\frac{1}{3}C⊗C^{-1}\right)=J^{-2/3}P^{T} \end{array},$$

where I denotes the fourth-order identity tensor

(18) $$I=\delta_{ik}\delta_{jl}e_{i}⊗e_{j}⊗e_{k}⊗e_{l},\;i,j,k,l=1,2,3$$

and $\delta_{ij}$ is the Kronecker delta function

(19) $$\delta_{ij}=\left\{\begin{array}{c} 1,i=j\\ 0,i\neq j \end{array}\right..$$

Note that the symbol ⊗ in Equation (14) and Equation (18) denotes a tensor product operator. The transpose of the fourth-order tensor $P^{T}$ in Equation (17) is the fourth-order projection tensor

(20) $$P=I-\frac{1}{3}C^{-1}⊗C.$$

In Equation (14), $C^{-1}$ is the inverse of C, and

(21) $$\begin{array}{cc} \; & \bar{S}=\frac{\partial \Psi_{\mathrm{iso}\;}}{\partial \bar{C}}=2\left[\frac{\partial \Psi_{\mathrm{iso}\;}}{\partial {\bar{I}}_{1}}\frac{\partial {\bar{I}}_{1}}{\partial \bar{C}}+\frac{\partial \Psi_{\mathrm{iso}\;}}{\partial {\bar{I}}_{2}}\frac{\partial {\bar{I}}_{2}}{\partial \bar{C}}\right]=2\frac{\partial \Psi_{\mathrm{iso}\;}}{\partial {\bar{I}}_{1}}I+2\frac{\partial \Psi_{\mathrm{iso}\;}}{\partial {\bar{I}}_{2}}\left[{\bar{I}}_{1}I-\bar{C}\right] \end{array}$$

is the modified second Piola–Kirchhoff stress tensor for nearly incompressible problems. The tensor I in the above equation is the second-order identity tensor defined as

(22) $$I=\delta_{ij}e_{i}⊗e_{j},\;i,j=1,2,3$$

Equation (14) is the complete expression of the isochoric second Piola–Kirchhoff stress tensor when the strain energy function Ψ depends on the isochoric principal invariants ${\bar{I}}_{a}$. Note that in Equation (15),

(23) $$p=\frac{\partial \Psi_{\mathrm{vol}}}{\partial J}$$

is the hydrostatic pressure. Note that the scalar *p* may only be determined through the equilibrium equations and the boundary conditions [13].

According to the above decomposition process, it can be seen that different forms of the strain energy function Ψ only change $\bar{S}$ and *p*. These stresses are key tensors in the subsequent process of deriving constitutive models using specific strain energy functions. Of course, the premise of determining these stress tensor expressions is to identify each specific strain energy function and then differentiate between them to determine the stress expression. At this point, a three-dimensional large deformation constitutive model framework has been constructed. However, since direct fitting in three-dimensional form is impossible during the parameter-fitting process, the constitutive model needs to be simplified to a one-dimensional form. Therefore, in the next section, we will derive the one-dimensional general solution form of the three-dimensional constitutive model. Since it is difficult to solve volumetric stress in the one-dimensional form directly, it can only be temporarily obtained using a non-direct method under a nearly incompressible framework. Previous studies [26] have shown that this simplification has little effect on the parameter–fitting accuracy.

### 2.4. Constitutive Relationship Under Three Deformation Modes

In this section, we derive stress expressions under three typical deformation modes: uniaxial (UT), planar (PT), and equibiaxial tension (ET). According to the definition of the biaxiality ratio $\mu =\frac{\ln\left(\lambda_{\min}\right)}{\ln\left(\lambda_{\max}\right)}$, the three deformation modes respectively correspond to $\mu^{\mathrm{UT}}=-0.5$, $\mu^{\mathrm{PT}}=0$, and $\mu^{\mathrm{ET}}=1$ [6]. The superscripts represent different deformation modes. The derivation in this section gives the one-dimensional stress expression in parametric form, which provides a theoretical basis for the subsequent parameter fitting.

#### 2.4.1. General Solution Form

Since the stresses in the three deformation modes are usually given in the form of the first Piola–Kirchhoff stress tensor *P* (nominal stress) during experiments, the general solution is given in the form of *P* below. Considering the principal direction deformation, the componential formulation of *P* can be expressed as

(24) $$P_{i}=\frac{\partial \Psi }{\partial {\bar{I}}_{1}}\frac{\partial {\bar{I}}_{1}}{\partial \lambda_{i}}+\frac{\partial \Psi }{\partial {\bar{I}}_{2}}\frac{\partial {\bar{I}}_{2}}{\partial \lambda_{i}}-\frac{1}{\lambda_{i}}p,\;i=1,2,3.$$

According to Equation (24), general expressions under the three deformation modes are

(25) $$\left\{\begin{array}{c} P_{1}^{\mathrm{UT}}=2\left[\frac{\partial \Psi }{\partial {\bar{I}}_{1}}+\frac{1}{\lambda }\frac{\partial \Psi }{\partial {\bar{I}}_{2}}\right]\left[\left[\lambda_{1}\right]-\frac{1}{{\left[\lambda_{1}\right]}^{2}}\right]\\ P_{1}^{\mathrm{PT}}=2\left[\frac{\partial \Psi }{\partial {\bar{I}}_{1}}+\frac{\partial \Psi }{\partial {\bar{I}}_{2}}\right]\left[\left[\lambda_{1}\right]-\frac{1}{{\left[\lambda_{1}\right]}^{3}}\right],\\ P_{1}^{\mathrm{ET}}=P_{2}^{\mathrm{ET}}=2\left[\frac{\partial \Psi }{\partial {\bar{I}}_{1}}+\frac{1}{\lambda }\frac{\partial \Psi }{\partial {\bar{I}}_{2}}\right]\left[\left[\lambda_{1}\right]-\frac{1}{{\left[\lambda_{1}\right]}^{4}}\right] \end{array}\right.$$

where subscripts 1 and 2 represent the loading directions. The methods for deriving the general expression under each of the three deformation modes are presented below.

1. Uniaxial Tension, UT
  The specimen is subjected to tensile loading exclusively along the 1-direction. Therefore, under the uniaxial tension mode, the deformation gradient $F^{\mathrm{UT}}$ and the right Cauchy–Green strain tensor $C^{\mathrm{UT}}$ are given by
  (26) $$F^{\mathrm{UT}}=\left[\begin{array}{ccc} \lambda & 0 & 0\\ 0 & \lambda^{-1/2} & 0\\ 0 & 0 & \lambda^{-1/2} \end{array}\right],\;C^{\mathrm{UT}}=\left[\begin{array}{ccc} \lambda^{2} & 0 & 0\\ 0 & \lambda^{-1} & 0\\ 0 & 0 & \lambda^{-1} \end{array}\right].$$
  where λ represents the stretch ratio in the 1-loading direction. The invariants of $C^{\mathrm{UT}}$ are defined as
  (27) $$I_{1}^{\mathrm{UT}}=2\lambda^{-1}+\lambda^{2},\;I_{2}^{\mathrm{UT}}=\lambda^{-2}+2\lambda ,\;\mathrm{and}\;I_{3}^{\mathrm{UT}}=1.$$
  Since the lateral contraction is free during uniaxial deformation, the first Piola-Kirchhoff stress tensor can be defined as
  (28) $$P^{\mathrm{UT}}=\left[\begin{array}{ccc} P_{1}^{\mathrm{UT}} & 0 & 0\\ 0 & P_{2}^{\mathrm{UT}}=0 & 0\\ 0 & 0 & P_{3}^{\mathrm{UT}}=0 \end{array}\right].$$
  Then, the hydrostatic pressure can be obtained as
  (29) $$p^{\mathrm{UT}}=\frac{2}{\lambda }\frac{\partial \Psi }{\partial I_{1}}+2\left[\lambda +\frac{1}{\lambda^{2}}\right]\frac{\partial \Psi }{\partial I_{2}}.$$
  Under uniaxial tension, the general solution for the stress is derived as follows:
  (30) $$P_{1}^{\mathrm{UT}}=2\left[\frac{\partial \Psi }{\partial I_{1}}+\frac{1}{\lambda }\frac{\partial \Psi }{\partial I_{2}}\right]\left[\lambda -\frac{1}{\lambda^{2}}\right].$$
2. Planar Tension, PT
  The specimen is subjected to tensile loading exclusively along the 1-direction. Therefore, under the planar tension mode, the deformation gradient $F^{\mathrm{PT}}$ and the right Cauchy–Green strain tensor $C^{\mathrm{PT}}$ are given by
  (31) $$F^{\mathrm{PT}}=\left[\begin{array}{ccc} \lambda & 0 & 0\\ 0 & 1 & 0\\ 0 & 0 & \lambda^{-1} \end{array}\right],\;C^{\mathrm{PT}}=\left[\begin{array}{ccc} \lambda^{2} & 0 & 0\\ 0 & 1 & 0\\ 0 & 0 & \lambda^{-2} \end{array}\right].$$
  The invariants of $C^{\mathrm{PT}}$ are defined as
  (32) $$I_{1}^{\mathrm{PT}}=I_{2}^{\mathrm{PT}}=\lambda^{2}+\lambda^{-2}+1,\;\mathrm{and}\;I_{3}^{\mathrm{PT}}=1.$$
  Since the contraction in the 3-direction is free during planar tension, the first Piola–Kirchhoff stress matrix can be written as
  (33) $$P^{\mathrm{PT}}=\left[\begin{array}{ccc} P_{1}^{\mathrm{PT}} & 0 & 0\\ 0 & P_{2}^{\mathrm{PT}} & 0\\ 0 & 0 & P_{3}^{\mathrm{PT}}=0 \end{array}\right].$$
  It should be noted that, considering the particularity of the planar tension deformation mode, there is no deformation in the 2-direction (approximately), and the constraint of the fixture causes $P_{2}^{\mathrm{PT}}\neq 0$. Moreover, $P_{2}^{\mathrm{PT}}$ is difficult to obtain directly through experiments. The hydrostatic pressure can, therefore, be obtained as
  (34) $$p^{\mathrm{PT}}=\frac{2}{\lambda^{2}}\frac{\partial \Psi }{\partial I_{1}}+2\left[1+\frac{1}{\lambda^{2}}\right]\frac{\partial \Psi }{\partial I_{2}}.$$
  Under planar tension, the general solution for the stress can be derived as
  (35) $$P_{1}^{\mathrm{PT}}=2\left[\frac{\partial \Psi }{\partial I_{1}}+\frac{\partial \Psi }{\partial I_{2}}\right]\left[\lambda -\frac{1}{\lambda^{3}}\right].$$
3. Equibiaxial Tension, ET
  The specimen is subjected to tensile loading exclusively along the 1- and 2-directions, with equal stretch ratios. Therefore, under the equibiaxial tension mode, the deformation gradient $F^{\mathrm{ET}}$ and the right Cauchy–Green strain tensor $C^{\mathrm{ET}}$ can be obtained as follows:
  (36) $$F^{\mathrm{ET}}=\left[\begin{array}{ccc} \lambda & 0 & 0\\ 0 & \lambda & 0\\ 0 & 0 & \lambda^{-2} \end{array}\right],\;C^{\mathrm{ET}}=\left[\begin{array}{ccc} \lambda^{2} & 0 & 0\\ 0 & \lambda^{2} & 0\\ 0 & 0 & \lambda^{-4} \end{array}\right].$$
  The invariants of $C^{\mathrm{ET}}$ are defined as
  (37) $$I_{1}^{\mathrm{ET}}=2\lambda^{2}+\lambda^{-4},\;I_{2}^{\mathrm{ET}}=2\lambda^{-2}+\lambda^{4},\;\mathrm{and}\;I_{3}^{\mathrm{ET}}=1.$$
  Since the contraction in the 3-direction is free during equibiaxial tension, the first Piola–Kirchhoff stress matrix can be written as
  (38) $$P^{\mathrm{ET}}=\left[\begin{array}{ccc} P_{1}^{\mathrm{ET}} & 0 & 0\\ 0 & P_{2}^{\mathrm{ET}} & 0\\ 0 & 0 & P_{3}^{\mathrm{ET}}=0 \end{array}\right].$$
  Then, the hydrostatic pressure can be obtained as
  (39) $$p^{\mathrm{ET}}=\frac{2}{\lambda }\frac{\partial \Psi }{\partial I_{1}}+2\left[\lambda +\frac{1}{\lambda^{4}}\right]\frac{\partial \Psi }{\partial I_{2}}.$$
  Under equibiaxial tension, the general solution for the stress can be derived as
  (40) $$P_{1}^{\mathrm{ET}}=2\left[\frac{\partial \Psi }{\partial I_{1}}+\frac{1}{\lambda }\frac{\partial \Psi }{\partial I_{2}}\right]\left[\lambda -\frac{1}{\lambda^{4}}\right].$$

#### 2.4.2. Specific Model

When developing a constitutive model, the key lies in determining its isochoric strain energy function $\Psi_{\mathrm{iso}}$, while the volumetric strain energy function $\Psi_{\mathrm{vol}}$ can be determined by setting a large bulk modulus to define its contribution. According to research, in order to determine the nonlinear large-deformation characteristics of elastometric materials, $\Psi_{\mathrm{iso}}$ needs to be a function of strain invariants as independent variables; to consider the biaxiality-ratio sensitivity, a second strain invariant must be introduced [27]. Therefore, we construct the following Carroll-like form [28] of the isochoric strain energy function

(41) $$\Psi_{\mathrm{iso}\;}=a{\bar{I}}_{1}+b{\bar{I}}_{1}^{4}+c\sqrt{{\bar{I}}_{2}},$$

where *a*, *b*, and *c* are material parameters. Based on the previous derivations, the general solutions for the stress expressions of Equation (41) under the three different deformation modes—uniaxial, planar, and equibiaxial tension—are, respectively, given as

(42) $$\left\{\begin{array}{c} P_{1}^{\mathrm{UT}}=\left[\begin{array}{c} 2a+8b{\left[2\lambda^{-1}+\lambda^{2}\right]}^{3}+c{\left[1+2\lambda^{3}\right]}^{-1/2} \end{array}\right]\left[\lambda -\lambda^{-2}\right]\\ P_{1}^{\mathrm{PT}}=\left[\begin{array}{c} 2a+8b{\left[\lambda^{2}+\lambda^{-2}+1\right]}^{3}+c{\left[\lambda^{2}+\lambda^{-2}+1\right]}^{-1/2} \end{array}\right]\left[\lambda -\lambda^{-3}\right]\\ P_{1}^{\mathrm{ET}}=P_{2}^{\mathrm{ET}}=\left[\begin{array}{c} 2a+8b{\left[\lambda^{-4}+2\lambda^{2}\right]}^{3}+c\lambda^{2}{\left[2\lambda^{-2}+\lambda^{4}\right]}^{-1/2} \end{array}\right]\left[\lambda -\lambda^{-5}\right] \end{array}\right..$$

Moreover, for the purpose of our subsequent comparative analysis, we also introduce several classic models below—the Neo-Hookean [15], Mooney–Rivlin model [16,17], Yeoh [29], and Ogden models [30]—and modify them based on our nearly incompressible framework.

1. The isochoric strain energy function of the Neo-Hookean model is given by
  (43) $$\Psi_{\mathrm{iso}\;}=\frac{c_{1}}{2}\left({\bar{I}}_{1}-3\right),$$
  where $c_{1}$ is the material shear modulus. Based on the previous derivations, the stress expressions under the three deformation modes can be derived as
  (44) $$\left\{\begin{array}{c} P_{1}^{\mathrm{UT}}=c_{1}\left[\lambda -\lambda^{-2}\right]\\ P_{1}^{\mathrm{PT}}=c_{1}\left[\lambda -\lambda^{-3}\right]\\ P_{1}^{\mathrm{ET}}=P_{2}^{\mathrm{ET}}=c_{1}\left[\lambda -\lambda^{-5}\right] \end{array}\right..$$
2. The isochoric strain energy function of the Mooney–Rivlin model is given by
  (45) $$\Psi_{\mathrm{iso}\;}=c_{10}\left({\bar{I}}_{1}-3\right)+c_{01}\left({\bar{I}}_{2}-3\right),$$
  where $c_{10}$ and $c_{01}$ are material parameters. Based on the previous derivations, the stress expressions under the three deformation modes can be derived as
  (46) $$\left\{\begin{array}{c} P_{1}^{\mathrm{UT}}=c_{10}\left[2\lambda -2\lambda^{-2}\right]+c_{01}\left[2-2\lambda^{-3}\right]\\ P_{1}^{\mathrm{PT}}=c_{10}\left[2\lambda -2\lambda^{-3}\right]+c_{01}\left[2\lambda -2\lambda^{-3}\right]\\ P_{1}^{\mathrm{ET}}=P_{2}^{\mathrm{ET}}=c_{10}\left[2\lambda -2\lambda^{-5}\right]+c_{01}\left[2\lambda^{3}-2\lambda^{-3}\right] \end{array}\right..$$
3. The isochoric strain energy function of the Yeoh model is given by
  (47) $$\Psi_{\mathrm{iso}\;}=\sum_{i=1}^{3}C_{i}{\left({\bar{I}}_{1}-3\right)}^{i},$$
  where $C_{i}$ is a material parameter. Based on the previous derivations, the stress expressions under the three deformation modes can be derived as
  (48) $$\left\{\begin{array}{c} P_{1}^{\mathrm{UT}}=\left[\begin{array}{c} 2c_{1}+4c_{2}\left[{\bar{I}}_{1}^{\mathrm{UT}}-3\right]\left.+6c_{3}{\left[{\bar{I}}_{1}^{\mathrm{UT}}-3\right]}^{2}\right]\left[\lambda -\lambda^{-2}\right] \end{array}\right.\\ P_{1}^{\mathrm{PT}}=\left[\begin{array}{c} 2c_{1}+4c_{2}\left[{\bar{I}}^{\mathrm{PT}}-3\right]+6c_{3}{\left[{\bar{I}}^{\mathrm{PT}}-3\right]}^{2} \end{array}\right]\left[\lambda -\lambda^{-3}\right]\\ P_{1}^{\mathrm{ET}}=P_{2}^{\mathrm{ET}}=\left[\begin{array}{c} 2c_{1}+4c_{2}\left[{\bar{I}}_{1}^{\mathrm{ET}}-3\right]+6c_{3}\left[{\bar{I}}_{1}^{\mathrm{ET}}-3\right] \end{array}\right]\left[\lambda -\lambda^{-5}\right] \end{array}\right..$$
4. The isochoric strain energy function of the Ogden model is given by
  (49) $$\Psi_{\mathrm{iso}\;}=\sum_{i=1}^{N}\frac{2g_{i}}{\alpha_{i}^{2}}\left({\bar{\lambda }}_{1}^{\alpha_{i}}+{\bar{\lambda }}_{2}^{\alpha_{i}}+{\bar{\lambda }}_{3}^{\alpha_{i}}-3\right),$$
  where $g_{k}$ and $\alpha_{k}$ are material parameters, *N* denotes the number of terms, and ${\bar{\lambda }}_{i}$ represents the equivalent principal stretch. Based on the derivation presented earlier, the stress expressions under the three deformation modes can be derived as
  (50) $$\left\{\begin{array}{c} P_{1}^{\mathrm{UT}}=\sum g_{k}^{\mathrm{UT}}\left[\lambda^{\alpha_{k}^{\mathrm{UT}}-1}-\lambda^{-\frac{1}{2}\alpha_{k}^{\mathrm{UT}}-1}\right]\\ P_{1}^{\mathrm{PT}}=\sum g_{k}^{\mathrm{PT}}\left[\lambda^{\alpha_{k}^{\mathrm{PT}}-1}-\lambda^{-\alpha_{k}^{\mathrm{PT}}-1}\right]\\ P_{1}^{\mathrm{ET}}=P_{2}^{\mathrm{ET}}=\sum g_{k}^{\mathrm{ET}}\left[\lambda^{\alpha_{k}^{\mathrm{ET}}-1}-\lambda^{-2\alpha_{k}^{\mathrm{ET}}-1}\right] \end{array}\right..$$

## 3. Experiments and Parameter Fitting

### 3.1. Experiments

**Figure 2:**
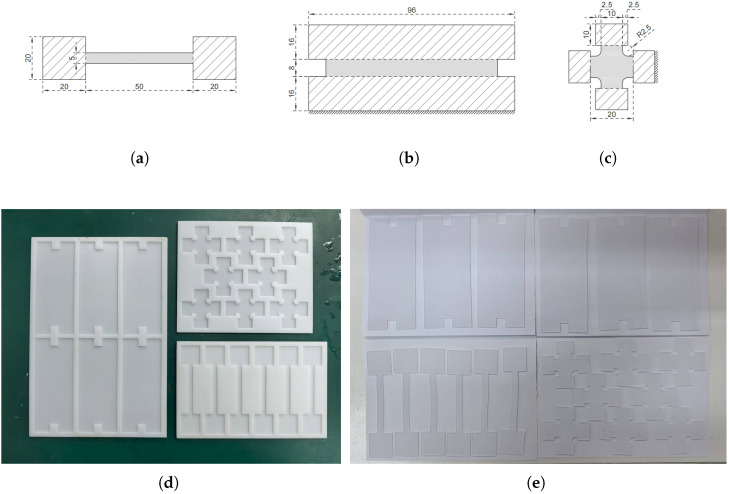
Dimensions of (**a**) uniaxial, (**b**) tension, and (**c**) equibiaxial tension specimens. (**d**) Moulds and (**e**) specimens.

The dimensions of the specimens are given in Figure 2a–c, each with a thickness of 1.5 mm. Ecoflex^TM^ silicone rubber was used in our experiments (00-20, 00-30, and 00-50); the material consists of two components, which were weighed to ensure a 1:1 mass ratio and manually mixed in a clean container until a homogeneous state was achieved. Subsequently, the mixture was poured into custom-made moulds (fabricated through resin polymer printing; see Figure 2d). Given that stirring introduces air bubbles, the mixture was then degassed in a vacuum chamber, typically for 5–10 min, until all visible bubbles were eliminated. Then, the specimens were left to cure at room temperature for at least 24 h to ensure complete crosslinking. A sample specimen is shown in Figure 2e.

**Figure 3:**
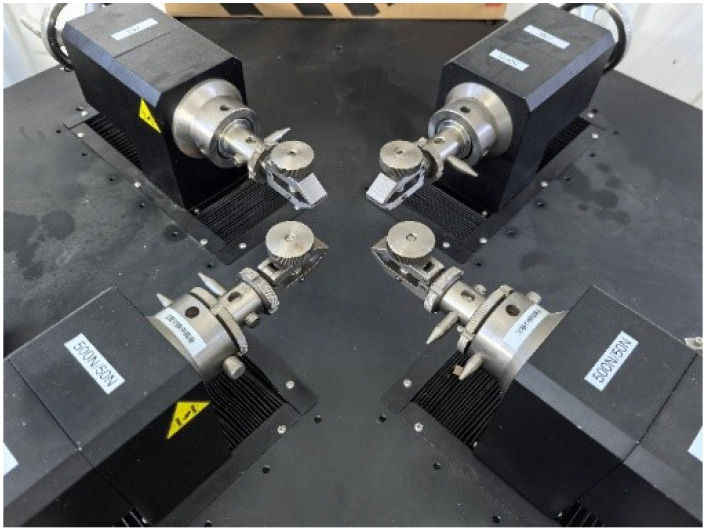
Testing machine.

**Figure 4:**
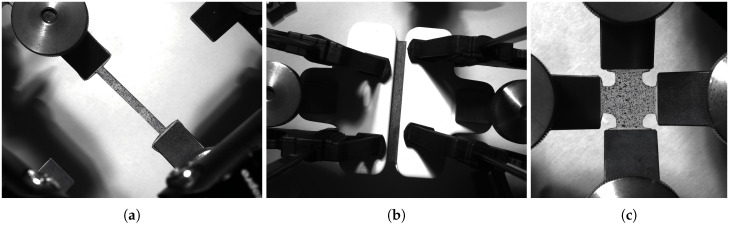
Application of clamping to (**a**) uniaxial, (**b**) planar, and (**c**) equibiaxial tension specimens.

All tests were conducted using a biaxial testing machine (see Figure 3, load cell with a capacity of 500 N). Each specimen was loaded at a quasi-static strain rate of 0.01/s under ambient laboratory temperature conditions. Note that the velocity dependence of the material was not taken into account in this study, as no significant disparity was observed in the stress values during pre-trial tests under different strain rates (0.001/s, 0.01/s, and 0.1/s). To prevent slippage during testing, aluminium tabs were bonded at the gripping locations. Four parallel trials were conducted for each test type, with specimens from the same batch used for each set of replicates to eliminate potential variations caused by the fabrication process. The nominal stress and strain of each specimen were calculated based on the recorded load-displacement history from the load cells. The testing machine was programmed to stop when specimen fracture occurs to thoroughly characterise failure behaviour. Prior to the test, speckled patterns were affixed to the surfaces of specimens to facilitate high-precision strain measurement using Digital Image Correlation (DIC). The DIC system utilised a camera sampling frequency of 1 Hz; the clamping photos are depicted in Figure 4.

**Figure 5:**
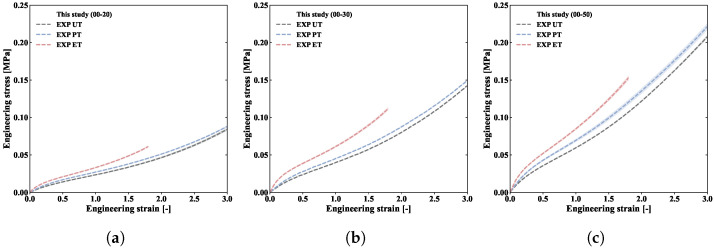
Engineering stress–strain curves for three deformation modes at Shore hardnesses of (**a**) 00-20, (**b**) 00-30, and (**c**) 00-50.

The engineering stress–strain curves are shown in Figure 5, where error bands representing the standard deviation are shown for each curve. It can be seen that these error bands are relatively small, indicating a high degree of consistency in the test results. The higher the biaxiality ratio (UT → PT → ET), the higher the stress–strain curve of the material. This suggests that the mechanical behaviour of silicone rubber is sensitive to biaxiality. The results presented in Figure 5 fully reflect this sensitivity, which has often been overlooked in previous studies. From a micro perspective, during deformation, the continuous internal rotation of the main chain and end sliding occur within the polymer, causing the molecular chains to change from a coiled to an oriented state, macroscopically corresponding to a large degree of deformation. Moreover, this orientation behaviour leads to hardening behaviour under loading, particularly in the equibiaxial tension curve. However, the molecular chain orientation patterns of each specimen under different biaxiality ratios are not consistent. As the ratio increases, the number of molecular-chain orientation directions increases. Since the chain segments in each orientation are not independent of each other, they synergistically strengthen the molecular chain network to resist external deformation, manifesting macroscopically as a higher stress response under higher deformation and biaxiality ratios.

Under various biaxiality ratios, differences in specimen failure can also be observed. Table 1 summarises the failure strains under different deformation modes at the 00-30 Shore hardness. The results show that as the biaxiality ratio increases, the failure strain decreases, indicating a relationship between the two. As previously discussed with regard to the microscopic mechanism, an increase in biaxiality ratio leads to orientation occurring in more directions, increasing the probability of disentanglement in the molecular chain network during deformation. When large-scale chain separation or covalent bond breakage occur, the polymer chain network separates, corresponding macroscopically to the material’s failure behaviour.

### 3.2. Parameter Fitting

The parameters for each model are fitted simultaneously by using the test data from all three deformation modes without applying weighting. Through the application of the least-squares method, the objective of the fitting procedure is to minimise the difference between the experimental stress–strain data and the predictions from the constitutive models via parameter adjustment. To evaluate the goodness of fit for each model, the root mean square error ($\mathrm{RMSE}=\sqrt{\frac{1}{n}\sum_{i=1}^{n}{\left(y_{i}-{\hat{y}}_{i}\right)}^{2}}$) is calculated, where $y_{i}$ represents the target values, ${\hat{y}}_{i}$ represents the predicted values, and *n* is the total number of data points. The fitting results are presented in Table 2.

**Figure 6:**
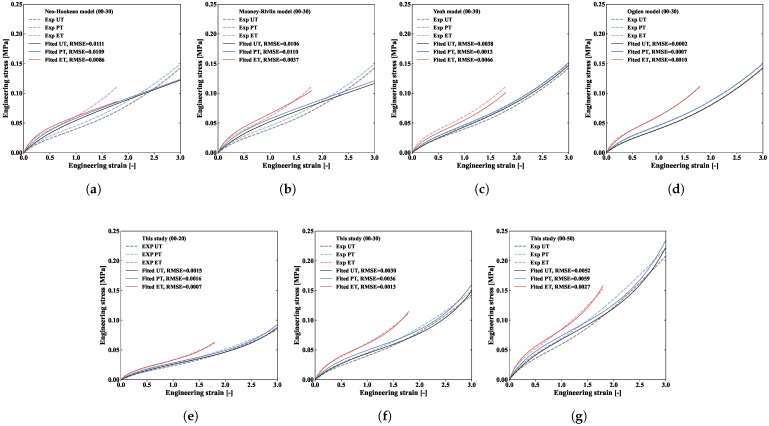
Fitting results of different constitutive models: (**a**) Neo-Hookean (00-30), (**b**) Mooney–Rivlin (00-30), (**c**) Yeoh (00-30), (**d**) Ogden (00-30), and ours ((**e**) (00-20), (**f**) (00-30), and (**g**) (00-50)).

The fitting results in Figure 6 demonstrate that different constitutive models describes mechanical behaviour differently under different stress–biaxiality ratios:

1. The Neo-Hookean (Figure 6a) and Mooney–Rivlin models (Figure 6b) both fail to accurately describe the mechanical behaviour under different biaxiality ratios, especially under equibiaxial tension conditions. This is because neither considers the material’s biaxiality-ratio sensitivity, and the Neo-Hookean model relies only on the first strain invariant, limiting its ability to describe complex deformation behaviours.
2. The Yeoh (Figure 6c), Ogden (Figure 6d), and proposed models (Figure 6e–g) demonstrate relatively good fitting performance. Both the Yeoh model and the proposed model contain three parameters; however, the proposed model achieves better fitting results with lower root mean square error (RMSE) values. The Ogden model, despite its higher fitting precision (lower RMSE), requires twice the number of parameters compared to the proposed model. Overall, the proposed model achieves a good balance between parameter number and fitting accuracy, demonstrating better engineering practicality.

Based on our fitting results, we discuss further the strategy for developing the proposed model. The isochoric energy is dependent on the sequential expansion of free energy to minimise the errors remaining in the stress responses of previous terms. The first term is constructed to identify stress–strain data associated with uniaxial tension. In accordance with the Neo-Hookean model, the term ${\bar{I}}_{1}$ is employed. However, according to the fitting results shown in Figure 6a, the Neo-Hookean model fails to capture the hardening behaviour of the material under uniaxial tension, especially in the large-deformation stage. Therefore, inspired by the Yeoh model in Figure 6c, a higher-order term ${\bar{I}}_{1}^{4}$ is introduced to capture this hardening behaviour. Furthermore, the fitting results of the Neo-Hookean model and the Yeoh model indicate that merely incorporating ${\bar{I}}_{1}$ is insufficient to describe the hardening behaviour of the ET curve. Thus, the second strain invariant term $\sqrt{{\bar{I}}_{2}}$ is introduced to capture this residual stress. This phenomenological strategy was initially proposed by Carroll [28], which we use to effectively determine the biaxiality-ratio sensitivity of elastomeric materials.

**Figure 7:**
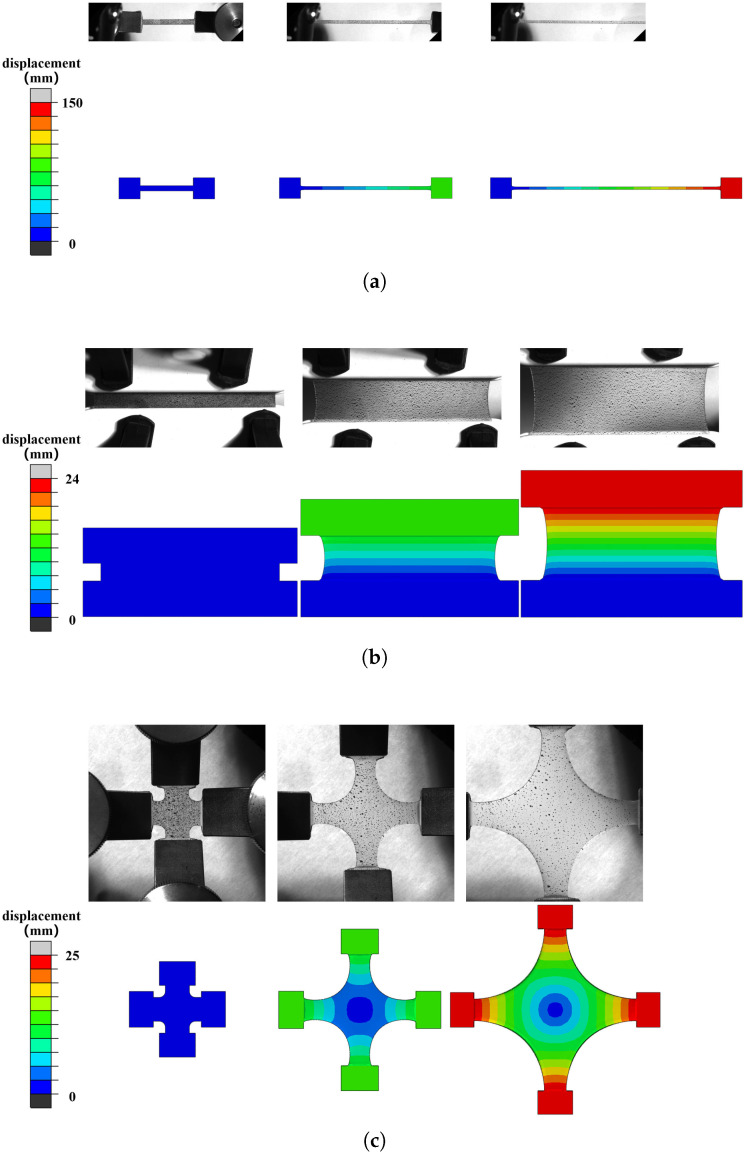
Comparison of deformation photos under (**a**) uniaxial, (**b**) planar, and (**c**) equibiaxial tension.

**Figure 8:**
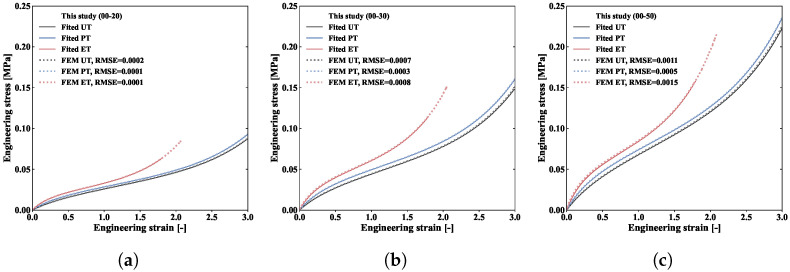
Comparison of fitted and simulated stress–strain curves at various Shore hardnesses, i.e., (**a**) 00-20, (**b**) 00-30, and (**c**) 00-50.

After parameter fitting, to further verify the accuracy of the proposed model, we conducted finite element simulations of uniaxial, planar, and equibiaxial tension tests with a mesh size of 1 mm. The specimen size, boundary conditions, and loading methods were consistent with our experiments, while the near-incompressible status of the material was reflected by setting a large bulk modulus of 1000 MPa. The deformation maps of our simulations and experiments are compared for the three modes in Figure 7a, Figure 7b, and Figure 7c, respectively, while the stress–strain comparison is shown in Figure 8. Overall, the constitutive model proposed in this paper can effectively describe the nonlinear large deformation and biaxiality-ratio sensitivity of silicone rubber materials. It should also be noted that no standardised stress calculation method currently exists for biaxial deformation, since it does not signify a uniform deformation condition. As such, our study integrates experiments, parameter fitting, and finite element simulations to iterate a correction factor for calculating biaxial stress at the specimen’s centre [31] in order to guarantee the accuracy of the calculation.

## 4. Conclusions

Silicone rubber materials have been widely applied in fields of engineering due to their excellent mechanical properties and chemical stability under complex deformation modes, which have attracted interest from both the academic and engineering communities.

In this study, we focused on developing a constitutive model for silicone rubber within the framework of continuum mechanics and an assumption of near incompressibility. Subsequently, uniaxial, planar, and biaxial tension experiments were systematically conducted at three Shore hardness levels; as the stress–biaxiality ratio and Shore hardness increased, the stress values of the material rose. Our experimental results exhibit high reproducibility (with relatively small error bands), indicating consistent and dependable experimental data. Our subsequent parameter calibration results indicate that this model can accurately and effectively describe the biaxiality-ratio sensitivity of silicone rubber materials (under the condition of 00-30 Shore hardness, the RMSE values for UT, PT, and BT were 0.0030 MPa, 0.0036 MPa, and 0.0013 MPa, respectively). Comparative analyses with four other classical hyperelastic constitutive models were also conducted, demonstrating the effectiveness and reliability of the proposed model. The Yeoh, Ogden, and proposed models all display good fitting performance, while the proposed model achieves a good balance between the number of parameters and fitting accuracy. After obtaining the model parameters, we performed finite element simulations of the uniaxial, planar, and biaxial tension tests. Our simulation results show good agreement with the experimental data in terms of deformation patterns and stress–strain curves. The maximum RMSE values between the simulated and fitted stress values were less than 0.0015 MPa across all deformation modes.

Overall, this research systematically integrates theoretical modelling, experimental testing, parameter fitting, and numerical simulation to explore the mechanical behaviour of silicone rubber material, especially its sensitivity to different deformation modes. Our approach and findings provide a reference for practical applications in sealing systems (e.g., optimising O-ring compression set performance under pressure loading) and soft robotics (e.g., enhancing the actuation precision of dielectric elastomer actuators). The limitations of this study are that the proposed model does not take into account the temperature effect, multiaxial fatigue behaviour, or anisotropic Mullins softening phenomena. These issues will be tackled in future research.

## Figures and Tables

**Table 1:** Failure strains under different deformation modes at a Shore hardness of 00-30.

Deformation Mode	UT	PT	ET
Failure strain	401%	324%	176%

**Table 2:** Fitting parameters of different constitutive models.

No.	Model Name	Shore Hardness	Parameter Value					
1	Neo-Hookean	00-30	$c_{1}$ [MPa] 3.100 × $10^{-2}$					
2	Mooney–Rivlin	00-30	$c_{10}$ [MPa] 1.467 × $10^{-2}$	$c_{01}$ [MPa] 5.115 × $10^{-4}$				
3	Yeoh	00-30	$c_{1}$ [MPa] 1.148 × $10^{-2}$	$c_{2}$ [MPa] 2.184 × $10^{-4}$	$c_{3}$ [MPa] 2.416 × $10^{-6}$			
4	Ogden	00-30	$g_{1}$ [MPa] 3.963 × $10^{-4}$	$g_{2}$ [MPa] 1.672 × $10^{-2}$	$g_{3}$ [MPa] 6.871 × $10^{-3}$	$\alpha_{1}$ [-] −2.690 × $10^{0}$	$\alpha_{2}$ [-] 1.400 × $10^{-1}$	$\alpha_{3}$ [-] 3.489 × $10^{0}$
5	This study	00-20	a [MPa] 7.077 × $10^{-3}$	b [MPa] 2.207 × $10^{-7}$	c [MPa] 1.644 × $10^{-3}$			
		00-30	a [MPa] 1.165 × $10^{-2}$	b [MPa] 3.905 × $10^{-7}$	c [MPa] 5.839 × $10^{-3}$			
		00-50	a [MPa] 1.869 × $10^{-2}$	b [MPa] 5.268 × $10^{-7}$	c [MPa] 3.173 × $10^{-3}$			

## Data Availability

Data will be made available on request from the corresponding author.

## Nomenclature

μ	biaxiality ratio
$\lambda_{i}$	principal stretch
Ψ	strain energy function
F	deformation gradient tensor
B	continuum body
*O*	origin of Cartesian coordinate system
$e_{i}$	basis vectors
*t*	time
$\Omega_{0}$	initial configuration
$\Omega_{t}$	current configuration
P	material particle
*X*	material point in the initial configuration
*x*	material point in the current configuration
*J*	determinant of the deformation gradient
*V*	initial volume of the differential element
*v*	current volume of the differential element
C	right Cauchy–Green deformation tensor
$F^{T}$	transpose of deformation gradient tensor
S	second Piola–Kirchhoff stress tensor
Θ	absolute temperature
*e*	internal energy per unit mass
Q	heat flux vector
P	first Piola–Kirchhoff stress tensor
$\bar{F}$	modified deformation gradient
$\bar{C}$	modified right Cauchy–Green strain tensor
$\Psi_{\mathrm{iso}}$	isochoric part of Ψ
$\Psi_{\mathrm{vol}}$	volumetric part of Ψ
${\bar{I}}_{1}$	first invariant of $\bar{C}$
${\bar{I}}_{2}$	second invariant of $\bar{C}$
$S_{\mathrm{iso}}$	isochoric part of S
$S_{\mathrm{vol}}$	volumetric part of S
I	fourth-order identity tensor
$C^{-1}$	inverse of C
$\bar{S}$	modified second Piola–Kirchhoff stress tensor
*p*	hydrostatic pressure
$\delta_{ij}$	Kronecker delta function
P	fourth-order projection tensor
$P^{T}$	transpose of P
$P_{i}$	principal stresses
